# COVID pandemic as an opportunity for improving mental health treatments of the homeless people

**DOI:** 10.1177/0020764020950770

**Published:** 2020-08-21

**Authors:** Carmen Martin, Pilar Andrés, Alberto Bullón, José Luis Villegas, Javier Ignacio de la Iglesia-Larrad, Berta Bote, Nieves Prieto, Carlos Roncero

**Affiliations:** 1 Psychiatric Service, University of Salamanca Health Care Complex, Salamanca, Spain; 2 Institute of Biomedicine of Salamanca (IBSAL), University of Salamanca, Salamanca, Spain; 3 Psychiatric Unit, School of Medicine, University of Salamanca, Spain

**Keywords:** Homeless COVID-19, prevention, psychiatric evaluation, rehabilitation

## Abstract

**Background:**

Homeless population has been severely affected by the COVID-19 pandemic. Their living conditions, comorbidity with different pathologies and a greater frequency of mental disorders, make this population vulnerable.

**Method:**

We implemented a program of serial visits in a hostel for confined homeless of the city council social services, for the monitoring and treatment of mental disorders and substance abuse problems. Accompanied by serial phone and email contacts.

**Results:**

A highly significant percentage (63%) had mental disorders or substance abuse, requiring pharmacological intervention, and 37% began follow-up in resources of the Mental Health and Addiction network of the Psychiatric Service at the end of the program. Hospital emergency service visits were drastically reduced. None of them were infected with COVID-19. An individualized Social plan was drawn up in order to reintegrate them with support in the community.

**Conclusions:**

The Results have been really positive, meeting all the objectives and opening up developing new programs in the future, in the pandemic outbreak and out of it.

## Highlights

- A high proportion of homeless people suffer from mental disorders, substance use disorders and comorbid medical pathologies.
- The current situation of the COVID 19 pandemic make them especially vulnerable to the possibility of infection and transmission of the illness.
- A group of patients has been assessed, avoiding the usage of the emergency services, and connecting them to the health care network.
- Neither the patients nor the professionals suffered COVID 19 contagion.

## Introduction

According to Global Homeless Statistics by the HWC in 2015, there are roughly 100 million homeless people (HP) in the world. In Spain, the National Integral Strategy for homeless people of 2015–2017 (Ministry of Health, Social Affairs and Equality) estimates that the number of HP is between 25,000 and 30,000, even though the numbers could be underestimated, given that HP who do not attend centers which could offer them different kinds of aid, and those who could be in a worse situation are not usually survey.

In Spain there has not been any global policy for homeless people which coordinates all the autonomic communities so far, which is why different professionals have pointed at the need for an integral strategy to establish a general framework to carry out such policies. By means of the [National Plan of Action towards Social Inclusion in the Kingdom of Spain] *(Plan Nacional de Acción para la Inclusión Social del Reino de España 2013–2016)* the Spanish government included the elaboration and activation of an Integral National Strategy for homeless people among the measures to perform, which states that such a process has to be carried out by all the involved agents together, including autonomous communities, local administrations and the social action sector.

Many factors contribute to such social and vital circumstances: domestic violence, unemployment, economic and family problems, childhood trauma, sexual assault, etc. (Habánik, 2018; Nishio et al., 2017). Among other factors, mental disorders play an important role, including psychotic disorders (Caton et al., 1994) and bipolar disorders (Maremmani et al., 2018). Fifty percent of HP have been considered to have some Kind of mental disorder (Scott, 1993). In 1 meta-analysis by Schreiter et al. (2017), in Germany, a prevalence of 77.4% was found for Axis I disorders, and 60.9% for substance use disorders, 55.4% related to alcoholism among them The presence of those disorders increases the risk of inability, medical comorbidity and mortality due to suicide (Hwang, 2000; Prigerson et al., 2003).

Dual disorders are present in aproximately 50% of the HP, with a prevlanece of 35% for mental disorders without substance use (Muñoz et al., 2004). The moment of apparition of mental disorders was reviewed within the study [“Intervention in homeless people with severe, chronic mental disorders in Europe”] (“*Intervención con personas sin hogar con trastornos mentales graves y crónicos en Europa”)* with respect to the moment of becoming homeless. It showed that first episodes of mental disorders and substance abuse were present before reaching the state of poverty. Even if no clear causal relationship can be established, mental disorders can be considered predisposing factors for the loss of home and social relationships.

The apparition of SARS-CoV-2 has caused a thorough reassessment of health care services for the whole global population, in different countries. Neto et al. (2020), describe the causes by which one of the groups most affected by the pandemic is that of HP, 1 of them being that their lack of accommodation is an obstacle for their isolation.

In the past, during the earlier pandemics of SARS and Influenza, it has been documented that the HP poses unique vulnerabilities to themselves and public health (Banerjee & Bhattacharya, 2020).

Homelessness makes these individuals more vulnerable for different reasons. First, worse health conditions, the lack of access to basic hygiene conditions like hand washing and to the use of disinfectant solution or masks increases the risk of contagion of the virus and its dissemination among the members of the closest group of contacts. The second relevant aspect is the lack of information due to the generally harder access to the media, necessary for learning the indications given by the government and for being updated on the situation surrounding the pandemic. Finally, aspects like malnourishment, depression and lack of sleep lead to an enfeeblement of the immune system, which could make these people prone to more severe symptoms of the illness (Lima et al., 2020) suffering from higher rates of chronic illnesses (Wood et al., 2020). Those who suffer from dual disorders and smoke also frequently suffer from lung diseases and are more vulnerable, with more complex necessities (Schütz et al., 2019). Because of all these reasons, HP younger than 65 have a mortality rate between 5 and 10 times higher than general population (Baggett et al., 2013) and the disease caused by COVID19 can make such mortality rates rise (Tsai & Wilson, 2020).

However, some studies have been published suggesting that psychopharmacologic agents could act as a protective factor against COVID (Plaze et al., 2020; Vaugeois, 2020). There is also some evidence supporting the use of antiretroviral drugs as therapeutic agents against the virus (Jiang et al., 2020). Both groups of drugs are usually prescribed in this population (Fazel et al., 2014; Hessol et al., 2019), which is why they could present some protective factors.

The Spanish National Statistics Institute points out that 24.3% of the Spanish HP and 75.7% HP coming from other countries do not have a health card which makes the access to health care services minimal, mainly because they get in contact with it through the emergency services (Hwang et al., 2013).

It must be remembered that there is a larger proportion of mental disease and substance abuse among this population, and self-observation in search of COVID19-related symptoms tends to be more lacking, as is the access to diagnostic tests. Furthermore, if the infection is confirmed, complying with the isolation standards is obviously impossible. There could be a greater resistance to being admitted to the hospital when it is necessary, which hardens the care process (Kar et al., 2020).

The aim of this work is to describe the health care and treatment process of mental health problems among HP in the city of Salamanca during the crisis caused by the COVID 19 pandemic.

## Material and method

Due to the state of alarm declared in Spain of March the 14th (BOE-A-2020-3692) (España, 2020), in order to managing the health care crisis situation caused by COVID 19, and which limits free circulation of people (Art. 7), HP have not been allowed to live on the street. Salamanca is located in Castile and Leon (Spain), The Salamanca province is inhabited by around 330,000 people and the city by 150.000 people and roughly 25.000–30.000 students (Junta de Castilla y León, 2020) who live there without being censed (Instituto Nacional de Estadística de España, 2020;Martín et al., 2019) (Basic data of the university system of Castilla y Leon 2019-2020). Social services in the Salamanca City Hall, in cooperation with NGO (such as Red Cros and Catholic Charity), has allowed HP to use the city’s municipal shelter, covering lodging and maintenance necessities as a consequence of the reduction in capacity of the preexisting resources.

Prior to the arrival of the pandemic, an integral assistance plan for HP had already been suggested, with the participation of the University of Salamanca Healthcare Complex (USHC) and the collaboration of the City Hall and NGOs of Salamanca, in order to bring a comprehensive assistance to this population, taking special care of the aspects related to mental health. The COVID pandemic was the trigger to start the chain of events that brought this program into action.

In this context, the Psychiatry Service in the (USHC) according to the contingency plan applied during the week of March the 16th 2020 (Roncero et al., 2020), initiated a program of primary care for the HP accepter in this center. This program is conceived with the aim of identifying mental disorders, problems related to substance abuse and intervening when necessary if psychopathological alterations or withdrawal symptoms were detected (due to the interruption or reduction of substance use during the lockdown period). The intention was to avoid the worsening of previously existing disorders, and which could make the cohabitation and lockdown as well as to prevent the usage of the emergency services during the most active period of the pandemic.

Seventy homeless people are believed to live in the city of Salamanca (Count of homeless of Salamanca city. Nov 2018) (City-hall of Salamanca, 2018). The HP who used this resource enabled for the pandemic were locked down in it willingly. Given the very specific kind of assistance that the patients were going to need, consisting in medical assistance and psychopharmacological interventions, the program was led directly by psychiatrists from the PD of the USHCC. Psychologists and social educators were part of the usual staff of the center, provided by the local city hall and NGOs.

The municipal shelter can normally accommodate anyone who respects the basic rules of cohabitation. It is especially aimed at students because it is located close to the university and to the municipal swimming pool which is frequently used by them during the summer. The shelter offers shared rooms for 2, 4 or 10 people, it has a buffet dining room for breakfast, lunch and dinner, and several shared areas inside and outside the building.

The HP who were living there were not allowed to leave the shelter because of confinement, they complied the same rules established by the government for the whole country. If they wanted to go to the streets, they had to justify their movements and ask for an authorization by the responsible authorities.

The first visit to the center takes place on March the 27th. Since then, our psychiatrists have kept attending the center every 8–10 days in pairs, carrying out a total of 7 visits in presence until May the 25th 2020. Given the higher risk among this population, and that their infective status was unknown, the interviews have been done with complete personal protective equipment (PPE), respecting the indications regarding social distance and preventive measures.

**Figure1.**
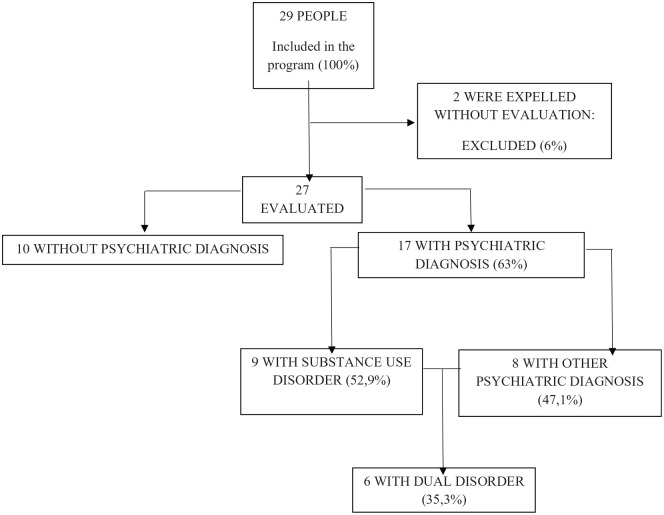
Sample flowchart.

During the first visit a general assessment was undertaken of all the users of the center, for an initial detection of possible cases and situation which needed intervention or follow-up. Twenty-two subjects were evaluated. During the following visits, already known patients were reassessed and new cases, people recently incorporated to the program and new situations which required intervention were also evaluated. The last visit was made on May 25 th 2020, therefore the length of the program was of 2 months. The resulting sample included 29 patients with 2 dropout instances (they left the shelter early after the beginning of lockdown (Figure 1).

During the time between interviews, 7 phone or mail contacts with the center managers were made to maintain a fluid communication to solve incidences and to inform about new cases or the state of the already known ones.

## Results

During the first visit, among 22 locked-down HP, 6 (27%) fulfilled DSM-5 criteria for some mental disorder and 5 (23.63%) presented symptoms without receiving a DSM-5 diagnose, but they were applied other measures: vigilance and follow-up, sleep hygiene and healthy lifestyle indications. Finally, 29 people were locked-down and 27 people among them were evaluated. There were no differences regarding demographic factors between HP with and without mental disorders (Table 1).

**Table 1.** Sociodemographic description of the evaluated population.

	Total N = 27	Mental health disorder N = 17	No mental health disorder N = 10	*p* Chi^2^
Gender	Male: 66.7%	64.4%	70%	.778
	Female: 33.3%	35.7%	30%	
Age in years. Average	37	39	34	.202
Place of Origin	Not Spanish: 22.2%	23.5%	20%	.417
	Other part of Spain: 33.3%	41.2%	20%	
	Salamanca: 44.4%	35.3%	60%	
Educational Level	No Primary Studies: 22.2%	23.5%	20%	.822
	Primary Studies: 59.3%	58.8%	60%	
	High School: 14.8%	11.8%	20%	
	University Studies: 3.7%	5.9%	0%	
Time Being Homeless	Less than 6 months: 51.9%	52.9%	50%	.145
	Between 6 months and 1 year: 7.4%	0%	20%	
	More than 1 year: 40.7%	47.1%	30%	
Employment Situation	Retired/Pensioner: 29.6%	29.4%	30%	.239
	Sick Leave: 3.7%	5.9%	0%	
	Unemployment: 59.3%	64.7%	50%	
	Active: 7.4%	0%	20%	
Legal Background	Yes: 22.2%	23.5%	20%	.831
Family Psychiatric History	Yes: 25.9%	35.3%	10%	.148
Other Medical History	Yes: 37%	35.3%	40%	.807

Three of them were already reported to have contacted psychiatry services previously, and almost 53% were under prescription for psychotropic drugs (mainly benzodiazepines and anti-depressants). A total of 35.3% of them suffered from other organic diseases as well. There are no gender differences regarding personal and family background of drug abuse and psychiatric history (Table 2)

**Table 2.** Gender differences among psychiatric patients.

	Total N = 17	**Gender:** Men: N = 11. Women: N = 6 *p* Chi^2^
Family Substance Abuse History	Yes: 29.4%	.726
Substance Abuse History	Yes: 52.9%	.232
Current Substance Abuse	No: 47.1%	.256
	Alcohol: 23.5%	
	Cannabis: 17.6%	
	Cocaine: 5.9%	
	Opiates/Methadone: 5.9%	
Dual Disorders	Yes: 35.2%	.90
Psychiatric Hospitalization History	Yes: 29.4%	.394

During the 7 times that the teams went to the shelter, 50 psychiatric visits were made. Ten people who had no mental disorder diagnosis were just visited once. There were 15 individuals who were only visited once (55.6%); 4 individuals who were visited twice (14.8%); 5 individuals who were visited 3 times (18.5%); and 3 individuals who were visited 4 times (11.1%), the global average per person being 1.85 visits. The average per person for those with a psychiatric diagnose was 2.35 visits. As well, 7 phone calls and 3 contacts per email were made by the psychiatric team to address the shelter and sociosanitary staff in order to detect and avoid relapses. Once the program had finished, mental disorders according DSM-5 criteria had been assessed in 63% of the sample (17), with a 53.9% (9) presenting substance use disorders and 47.1% presenting another kind of mental disorders: 29.4% being anxiety disorders (5) 11.7% affective disorders (2) and 17.6% being labeled as a psychotic disorder (1). Six patients (35.3%) are considered dual disorder patients.

At the start of the program 58.8% of the patients received psychotropic drugs, and at the end 82.3% of them had prescribed (*p*: 0.04). Antipsychotics were used in 29.4% of the people, antidepressants in 28.5% and benzodiazepines in 23.5%. The visits to the emergency service at the hospital by this group decreased from 23.6% to a 5.9%, given that only one patient used them once during this time, motivated by a clinically relevant cause, with the suspicion of a possible CVA. (Table 3).

**Table 3.** Comparative regarding treatment prescription before and after starting the program.

Main psychiatric treatment	Before psychiatric visit	After psychiatric visit	*p* Chi^2^	Attended psychiatric emergencies in the previous 2 month	Attended psychiatric emergencies during pandemic	*p* Chi^2^	Follow-up by mental health before pandemic	Follow-up by mental health after pandemic	*p* Chi^2^
Yes	58.8%	82.3%	.043	23.6%	5.9%*	0.063	17.7%	52.9%	0.072
No	41.3%	17.3%		76.4%	94.1%		82.2%	47.1%	
Antipsychotics:	11.8%	29.4%	.020						
Benzodiazepines:	23.5%	23.5%	–						
Antidepressants:	23.5%	29.4%	.01						
Mood Stabilizers:	0%	0%	–						

* Before the program started.

When the program ended, all the users left the resource. Among the patients identified, 10 were referred to mental health resources: 4 to outpatient mental health teams and 3 to the outpatient drug clinic of the PS, 2 patients to others Outpatient Drug Clinics and 1 patient was referred to a therapeutic community for drug cessation and rehabilitation as a treatment of their substance use disorder. From the social point of view and with the aim of keeping them from reaching their precarious situation from before lockdown, professionals from the center created an individualized plan with each of the users to support them to reintegrate within the community. Of the 17 users who presented some psychiatric diagnosis, 5 have been referred to a Day Center for a long term stay and have been linked to mental health resources. One patient was referred provisionally to the same shelter until she accessed a release salary and acquired the means necessary to rent a home of his own. Other patients were referred to a shelter house for people living with HIV/AIDS. The rest of the users with a psychiatric diagnosis were assisted with the application for the minimum guaranteed income, house rental and search of employment, facilitating their travel to their place of work if needed. 9 out of 10 patients without a psychiatric diagnose were given support to apply for the minimum guaranteed income and 1 refuses all support.

Six weeks after the center’s closure, we contacted the referral professionals of the 10 patients who were referred to mental health and drug devices. The 3 patients referred to the mental health teams have been reviewed by the corresponding psychiatrists and 1 of them has also received psychological support, remaining stable. One of the 3 patients referred to the outpatient drug clinic of de PS, has abandoned treatment and refused follow-up, the other 2 remain abstinent and stable from a psychopathological point of view. One of the 2 patients referred to others Outpatient Drug Clinic remains stable; 1 has suffered a relapse of cocaine use and is awaiting admission to the Inpatient Detoxification Unit of the PS. The patient who was referred to a therapeutic community, asked for voluntary discharge after 10 days of treatment.

None of the locked-down users in the center suffered from COVID 19 disease. Twenty-seven patients underwent rapid total antibodies testing, all of them with negative results. Two patients were tested with polymerase chain reaction tests, 1 of them being admitted to the Salamanca University Hospital in the Internal Medicine ward due to compatible respiratory symptoms for a short time, where a COVID 19 disease was ruled out. The other PCR was carried out in the context of the TC referral protocol. The psychiatrists involved in the program were not infected either.

## Discussion

Homeless population received direct assistance during the pandemic and their contagion was avoided. More than 60% of them presented mental disorders and within 8 weeks they were visited in person 2–3 times. There are differences between treatments prior to and after the intervention, and the contact with the emergency services in the hospital was avoided, which could have contributed to none of them getting infected. Finally, 51.8% were linked to social and health care services and 37% to mental health resources, which can constitute a step forward in their reintegration and normalization.

The prevalence of mental disorders was 63%, being substance use disorders the most frequent diagnose (33%). This prevalence is found within the usually described range (Schreiter et al., 2017; Scott, 1993), except for psychotic disorders: Ayano et al. (2019), describe that 21% of the HP present psychotic disorders, in our sample there were only 4% of them. It is to be noted that all the patients sheltered in this resource entered it voluntarily. This circumstance can be a source for bias regarding the prevalence assessed in this study (Fazel et al., 2014). Moreover, the exceptional of the situation had to be considered, since usually people living with mental disorders or suffering from substance use disorders lack insight regarding their illness (Amador et al., 1991; Arango et al., 2011) and therefore it can be theorized that in a different context it would have been difficult for them to accept the offer of help that they were given in our program. Around 20% of the sample were patients with dual disorders, which was expectable considering other previously described populations (Doré-Gauthier et al., 2019; Jiménez-Castro et al., 2011). In this population a relationship between craving and impulsivity has been described, these aspects should be considered as main factors for their treatment (Robles-Martínez et al., 2019).

The presence of a masculine majority, without sociodemographic differences in the clinical variables when compared to women, is also found within the expectable. There are no clinical differences between people suffering from a mental disorder and those who do not. However, it must be noted that the sample is limited and the generalization of these conclusions has to be made cautiously.

Prescribed drugs are destined mainly to treat withdrawal, insomnia and anxiety symptoms, hence the pharmacological strategies used are based on antipsychotics and benzodiazepines, as a usual treatment for such symptoms, and antidepressants for those cases in which anxious-depressive or adaptative disorders were also present. There are some differences in the increase of the number of drug prescriptions made under this program, which were effective, given that there were no clinically complex situations or instances of severe relapse and the usage of the emergency services was avoided. That is greatly relevant, in the first place because health care authorities insisted reducing the number of visits to the emergency room except in case of extreme when strictly necessary, avoiding the risk of contagion and keeping them from collapse, because if emergencies in those patients are usually conflictive (Moore and Rosenheck, 2016; Salhi et al., 2018), they could be even more so during the pandemic, resulting in important risks for individual and public health and in a risk of infection by COVID 19 (Tsai & Wilson, 2020). Two to three visits were made per diagnosed patient, which add to communications established by phone with the professionals responsible for the center, all of which constitutes an intensive intervention that could explain the lack of violent incidents described in this population (Fernández-García-Andrade et al., 2020).There are few detection and early intervention programs for HP populations and, to our knowledge, the evaluation of such programs during the COVID 19 pandemic has not been documented. During this period, the access to sociosanitary services should be ensured for this population, besides controlling risks. In this sense, we think that programs like the one we have designed by our service in Salamanca are opportune and necessary. We have given support to mental health issues detected within this population, avoiding the apparition of severe psychopathological symptoms and the exacerbation of pre-existing mental illness in already diagnosed patients, which made their cohabitation easier in the center. Thus, in addition to their wellbeing, visits to the emergency services have been avoided, which were the main objectives behind the creation of the program. Also, thanks to the implementation of the program, 17 new patients have been identified who, after the program ended, have been integrated with the community mental health services via referrals to their mental health teams and the addictive disorders unit and other resources in the addiction care network (outpatient drug clinics and therapeutic communities) (Volkow, 2020), which leads to their reincorporation to society, as it has been proposed (Fazel et al., 2014). There are previous experiences which suggest that damage reduction programs and low threshold programs are useful to supply patients with basic resources which could be an entry point to the general and mental health care system (Martínez-Luna et al., 2018) and are well perceived by the patients, some of them in a vulnerable situation or homeless (Daigre et al., 2010).

The 17 patients presenting psychiatric disorders have been provided with economic resources and a place to live, in addition to treatment which is fundamental for their evolution since solving these problems is fundamental for their integration (Stergiopoulos et al., 2020). In this sense, it has been described that referring them to social services and providing them with a place to live, in addition to mental health interventions, is associated with an improvement in their functioning and severity of their disorders (Doré-Gauthier et al., 2019). However, the homeless and mental health topic needs advanced research to explore psychosocial and epidemiological correlates for preventing, identifying and treating mental disorders among homeless populations (Hossain et al., 2020), in the low-income patients (Neto et al., 2020) and in the European Countries.

Chronic mental health problems like stigma and isolation may arise from the social and psychological impact of COVID-19, which very likely remains largely unnoticed by the general public due to the attention and effort put into avoiding transmission (Torales et al., 2020). Strengths and weaknesses

Participation in this program has been voluntary, which is the reason why it is possibly not representative of the whole homeless population, and the sample is small, although, taking the size and population of the city into consideration, it comprise half of the homeless population living in it. The study must be considered because it reflects the assistance and development of a real-world program in an extreme situation and can lead to the preparation of future programs either during a new pandemic or not.

## Conclusion

Due to the pandemic caused by COVID 19, social and health care circumstances relative to homeless population, which usually are complicated, could be even more difficult. However, due to the intervention and implementation of this new program in the City Hall resources made by the Psychiatry Service, the objectives of detecting, treating and referring patients to social and mental health care resources, turning the unfortunate situation of the pandemic into an opportunity for this population.

For mental healthcare professionals and specialists of other areas this program shows that in many occasions, secondary and primary prevention can be very useful, avoiding risks for individual and public health. This suggests that we must be especially proactive and that such programs must be implemented during the crisis and out of them.
